# Long-Term Correction of Murine Glycogen Storage Disease Type III by AAV-Mediated Gene Therapy Using an Immunotolerizing Dual Promoter to Express Bacterial Pullulanase

**DOI:** 10.1155/acg2/4639392

**Published:** 2025-04-17

**Authors:** Kuo-An Liao, Jeong-A Lim, Su Jin Choi, Haiqing Yi, Baodong Sun

**Affiliations:** Division of Medical Genetics, Department of Pediatrics, Duke University School of Medicine, Durham, North Carolina, USA

**Keywords:** AAV gene therapy, glycogen storage disease type III, immunotolerizing dual promoter, long-term efficacy, pullulanase

## Abstract

**Background:**

We recently reported an innovative gene therapy approach for GSD III using a recombinant adeno-associated virus serotype 9 vector (AAV9-Dual-Pull) expressing a bacterial debranching enzyme (pullulanase) driven by a tandem dual promoter that consists of an immunotolerizing liver-specific promoter (LSP) and the ubiquitous CMV enhance/chicken *β*-actin (CB) promoter. In this follow-up study, we evaluated the long-term efficacy of this gene therapy in GSD IIIa mice.

**Methods:**

Three-month-old GSD IIIa mice were intravenously injected with AAV9-LSP-Pull or AAV9-Dual-Pull at the same dose (2.5 × 10^13^ vg/kg). Tissues were collected after 9 months for AAV genome quantification, pullulanase expression determination, and glycogen content measurement. Liver and muscle enzymes in plasma and disease biomarker in urine were analyzed at multiple time points to examine the correction of liver and muscle damage. Behavioral tests were performed during the course of AAV treatment to evaluate the improvement of muscle function.

**Results:**

The AAV-Dual-Pull treatment led to persistent pullulanase expression and effective glycogen reduction in the liver, heart, and skeletal muscle, accompanied by the reversal of liver fibrosis, decrease of plasma enzyme activities, and long-term improvement of muscle function. The AAV-LSP-Pull treatment showed a better therapeutic efficacy in the liver but had no effect on the cardiac and skeletal muscles.

**Conclusion:**

Our results demonstrated the long-term efficacy and safety of systemic AAV9-Dual-Pull delivery in GSD IIIa mice. Future studies will test this gene therapy approach in GSD IIIa dogs prior to the clinical translation to GSD III patients.

## 1. Introduction

Glycogen storage disease type III (GSD III, also known as Cori’s disease) is an autosomal recessive disorder caused by pathogenic mutations in the *AGL* gene coding for the glycogen debranching enzyme (GDE), a bifunctional enzyme with two independent catalytic activities, 4-a-D-glucanotransferase (EC 2.4.1.25) and amylo-a-1,6-glucosidase (EC 3.2.1.33). GDE is a key enzyme in glycogenolysis, which acts along with glycogen phosphorylase to release glucose-1-phosphate and glucose from glycogen branches. Deficient activity of GDE leads to impaired glycogen debranching and increased accumulation of abnormally structured glycogen (limit-dextrin) in liver, heart, and skeletal muscle. Two major GSD III subtypes based on the extent of the tissue involvement have been identified: most individuals (~85%) exhibit both liver and muscle involvements (GSD IIIa), while others (~15%) present symptoms only in the liver (GSD IIIb). Selective loss of GDE glucosidase activity (GSD IIIc) or glucanotransferase activity (GSD IIId) has been reported in extremely rare cases [1–3].

GSD III is a biphasic disorder with variable clinical presentations. In infancy and early childhood, the common features of GSD III are mostly liver involvements, including hepatomegaly, hypoglycemia, hyperlipidemia, and elevated hepatic transaminases. In adolescence and adulthood, liver symptoms become less pronounced, but progressive liver fibrosis can occur, and some patients may continue to develop long-term complications such as liver cirrhosis and hepatocellular carcinoma [3, 4]. Skeletal and cardiac muscle diseases appear during childhood and are the major causes of significant mobility and modality in adult patients. Muscle weakness and myopathy progressively develop with age, and most patients with GSD IIIa exhibit exercise-induced muscle pain and exercise intolerance [3, 5]. Cardiac involvement is frequent in GSD IIIa; most patients display left vertical hypertrophy, and some have asymptomatic/symptomatic cardiomyopathy. In severe cases, sudden death caused by cardiac failure and arrhythmias has been reported [3, 6].

Currently, there is no curative treatment for GSD III. The only therapeutic option is through symptomatic dietary management with uncooked starch and a high-fat high-protein diet. Although the dietary treatment seemed to be effective in ameliorating hypoglycemia and stabilizing cardiac manifestation [7–9], its beneficial effect on skeletal muscle remains unclear. Enzyme replacement therapy with recombinant human acid alpha-glucosidase (GAA) has been developed to treat Pompe disease (GSD II) [10]. However, this approach is not feasible for treating GSD III due to the lack of a natural receptor-mediated uptake of the therapeutic GDE from the blood into target tissues. Considering the unmet medical need and the monogenic nature of GSD III, gene replacement therapy with adeno-associated virus (AAV) vectors, particularly AAV serotype 9 (AAV9), is an optimal treatment approach for GSD III because AAV vectors can reliably transduce both liver and muscle tissues. Furthermore, numerous preclinical studies in multiple animal models and clinical trials have established AAV’s safety and efficacy profiles [11–19]. In the past decade, AAV has become the most common gene delivery vector in clinical trials for a broad range of human genetic diseases, especially disorders of inborn errors of metabolism [20]. However, the large size of human GDE cDNA (4.6 kb) prevents the efficient encapsidation of AAV, whose carrying capacity is less than 4.7 kb. This major limitation hinders the development of an effective AAV-mediated gene therapy for GSD III.

To overcome this issue, our laboratory has recently developed an innovative AAV gene therapy approach using a bacterial GDE, pullulanase, whose cDNA size (2.2 kb) is small enough for efficient AAV packaging. Injection of an AAV vector carrying a codon-optimized pullulanase cDNA driven by the ubiquitous CMV enhancer/chicken *β* actin promoter (AAV-CB-Pull) resulted in persistent transgene expression and glycogen correction in heart and skeletal muscle in infant GSD IIIa mice [21]. However, this vector triggered transgene-induced cytotoxic T lymphocyte (CTL) response in adult GSD IIIa mice, leading to a rapid loss of pullulanase expression in AAV-transduced tissues [22]. Immune responses against bacterial enzymes like Cas9 have been reported in several gene editing studies [23, 24]. Liver-directed AAV gene therapy using a liver-specific promoter (LSP) is a proven approach to induce immune tolerance to therapeutic transgene products [25, 26]. Based on this notion, we developed a tandem LSP-CB dual promoter by incorporating an LSP into the original AAV-CB-Pull plasmid before the CB promoter, generating the AAV-Dual-Pull vector. Administration of the AAV-Dual-Pull vector in adult GSD IIIa mice significantly decreased pullulanase-induced CTL response, resulting in widespread pullulanase expression and glycogen reduction in liver and muscles, reversal of liver fibrosis, and correction of disease phenotypes after 10 weeks [22].

In this study, we aimed to examine the long-term efficacy of the AAV-Dual-Pull treatment in adult GSD IIIa mice.

## 2. Materials and Methods

### 2.1. AAV Viral Packaging and Viral Vector Administration to Mice.

The pAAV-LSP-Pull and pAAV-Dual-Pull vectors were packaged in AAV9 as described [22]. The titer of the viral stock was determined by Southern blot using purified viral DNA and a biotin-labeled probe generated with Prime-A-Gene labeling kit (Promega, Madison, Wisconsin). The viral vector stock was handled according to Biohazard Safety Level 2 guidelines published by the National Institutes of Health. Three-month-old GSD IIIa (*Agl* knockout) mice [21] were injected with AAV9-LSP-Pull or AAV9-Dual-Pull at the same dose of 2.5 × 10^13^ vg/kg. After 9 months, the mice were sacrificed to collect tissues following overnight fasting. Gender- and age-matched untreated (UT) GSD IIIa mice and wild-type (WT) mice were used as controls. Fresh tissue specimens were either immediately frozen on dry ice and stored at −80°C until used for biochemical analyses or fixed immediately for histology.

### 2.2. AAV Vector Biodistribution.

AAV vector genomes in tissues were quantified by real-time PCR. Genomic DNA was extracted from frozen tissues using the Wizard Genomic DNA Purification kit (Promega, Madison, Wisconsin). PCR was performed using SYBR Green (Roche, Basel, Switzerland) and the gene-specific primer pairs for pullulanase and mouse *β*-actin as described [22]. The linearized pAAV-LSP-Pull plasmid DNA was used to generate a standard curve for calculating viral vector copy numbers.

### 2.3. Pullulanase Activity Assay.

Pullulanase activity was measured using the pullulanase activity assay kit (Megazyme, Wicklow, Ireland) as described [22]. Protein concentrations in tissue lysates were determined by BCA assay and used to normalize the data.

### 2.4. Immunohistochemistry (IHC).

Paraffin-embedded sections were deparaffinized and rehydrated. The slides were incubated in Tris-EDTA (pH 9.0) buffer at 100°C for 20 min for antigen retrieval and then washed with cold tap water and TBS containing 0.025% Triton X-100 (TBST). The samples were incubated with 10% normal goat serum with 1% BSA in TBS for 2 h at room temperature for blocking and with anti-HA antibody (Cell Signaling Technology, Danvers, Massachusetts) diluted in 1% BSA/TBS at 4°C overnight to detect pullulanase. The next day, the slides were washed with TBST, incubated with 0.3% H_2_O_2_ in TBS for 15 min, and incubated with HRP conjugated secondary antibody for 1 h at room temperature. The samples were washed and developed with SignalStain DAB Substrate Kit (Cell Signaling Technology, Danvers, Massachusetts). The slides were rinsed, counterstained with hematoxylin, dehydrated, cleared, and mounted. Images were taken on a BZ-X710 microscope (Keyence America, Itasca, Illinois).

### 2.5. Glycogen Content Assay.

Glycogen contents in the tissues were measured as described [22] with some modifications. Briefly, the tissues were homogenized in distilled water (1 mg of tissue/20 *μ*L of water) using a homogenizer, followed by sonication for 1 min (5-s pulse and 5-s rest cycle) with 30% amplitude (Qsonica, Q500A, Newtown, Connecticut). The lysates were diluted (1:5) in distilled water and boiled for 3 min to inactivate endogenous enzymes. The diluted samples were incubated with 0.175 U/mL (final concentration in the reaction) of amyloglucosidase (Sigma-Aldrich Co., St. Louis, Missouri) for 90 min at 37°C. The reaction mixtures were then boiled again for 3 min to stop the reaction. Thirty microliters of the mixtures was incubated with 1 mL of Pointe Scientific Glucose (Hexokinase) Liquid Reagents (Fisher, Hampton, New Hampshire) for at least 10 min at room temperature. The absorbance at 340 nm was read using a UV-VIS spectrophotometer (Shimadzu UV-1700 PharmaSpec, Tokyo, Japan).

### 2.6. Histology.

Fresh tissues were fixed immediately in 10% neutral-buffered formalin (NBF) for 48 h. After primary immersion fixation, the samples were postfixed with 1% periodic acid (PA) in 10% NBF for 48 h at 4°C. The samples were then washed with PBS, dehydrated with ascending grades of alcohol, cleared with xylene, and infiltrated with paraffin. For PAS staining, sections of paraffin-embedded tissues were processed and stained using Schiff reagent as described [21]. For trichrome staining, the paraffin-embedded liver sections were processed and stained using Masson’s trichrome staining kit (Sigma-Aldrich Co., St. Louis, Missouri), following the manufacturer’s protocol. Images were taken on a BZ-X710 microscope (Keyence America, Itasca, Illinois). The density of fibrotic tissues in the liver was measured using ImageJ software as described [22].

### 2.7. Plasma Enzyme Activity Assays.

Whole blood was collected in a green blood collection tube (coated with lithium heparin). The plasma was separated by centrifugation at 2000 × *g* at 4°C for 10 min and then diluted (1:5) with saline (0.9% (*w*/*v*) of NaCl). The activities of plasma alanine aminotransferase (ALT), aspartate aminotransferase (AST), and creatine kinase (CK) were measured using the Liquid ALT (SGPT), Liquid AST (SGOT), and Liquid CK Reagent Set, respectively (Pointe Scientific, Inc., Canton, Michigan) following the manufacturer’s protocol.

### 2.8. Determination of Urinary Glc4 Concentration.

The concentration of urinary Glc4, a glucose tetrasaccharide elevated in untreated GSD IIIa mice, was tested by stable isotope-dilution electrospray tandem mass spectrometry as described [27].

### 2.9. Behavioral Tests

#### 2.9.1. Treadmill Exhaustion Test.

Mice were acclimated for 15 min in the chamber of the treadmill (LE8709, Panlab Harvard Apparatus, Holliston, Massachusetts) and warmed up by running at the lowest speed (5 cm/s) and 25° of slope for 3 min. Then, mice were allowed to run at 8 cm/s for 3 min. The speed was increased by 4 cm/s every 3 min until the mice were exhausted or the maximal speed (32 cm/s) was reached.

#### 2.9.2. Wire Hang Test.

Mice were put on the center of a wire mesh positioned 30 cm above soft bedding. The wire mesh was carefully inverted, and the time until the mouse fell or the maximum time (3 min) was recorded. Three separate trials were recorded, and the maximum hanging time was used for comparison.

#### 2.9.3. Grip Strength Test.

The grip strength for four limbs was assessed by the grip test equipment (BIOSEB, Pinellas Park, Florida, BIO-GS3) following the manufacturer’s protocol. Briefly, put the mouse on the grid horizontally and allow it to grasp the grid using its four paws. Pull the mouse back by its tail while ensuring the body remains parallel with the grid, and record the maximal value displayed on the screen. Record the data from three separate trials and normalize the average force measurement by body weight.

### 2.10. Statistical Analysis.

All the data presented in this manuscript are shown as mean ± standard deviation (SD). Statistical significance was determined by unpaired Student’s *t*-test and ordinary one-way and two-way ANOVA with Tukey’s post hoc correction using GraphPad Prism software. *p* values < 0.05 were considered significant.

### 2.11. Study Approval.

The present studies in animals were reviewed and approved by Duke University Institutional Animal Care and Use Committee (Approval Number A010-23-01) (Durham, North Carolina). All animal care and experiments were conducted in accordance with approved institutional guidelines and the guidelines from the National Institute of Health guide for the care and use of laboratory animals.

## 3. Results

### 3.1. Intravascular Administration of AAV-Dual-Pull in GSD IIIa Mice Resulted in Persistent Pullulanase Expression in Major Affected Tissues.

**Figure 1:**
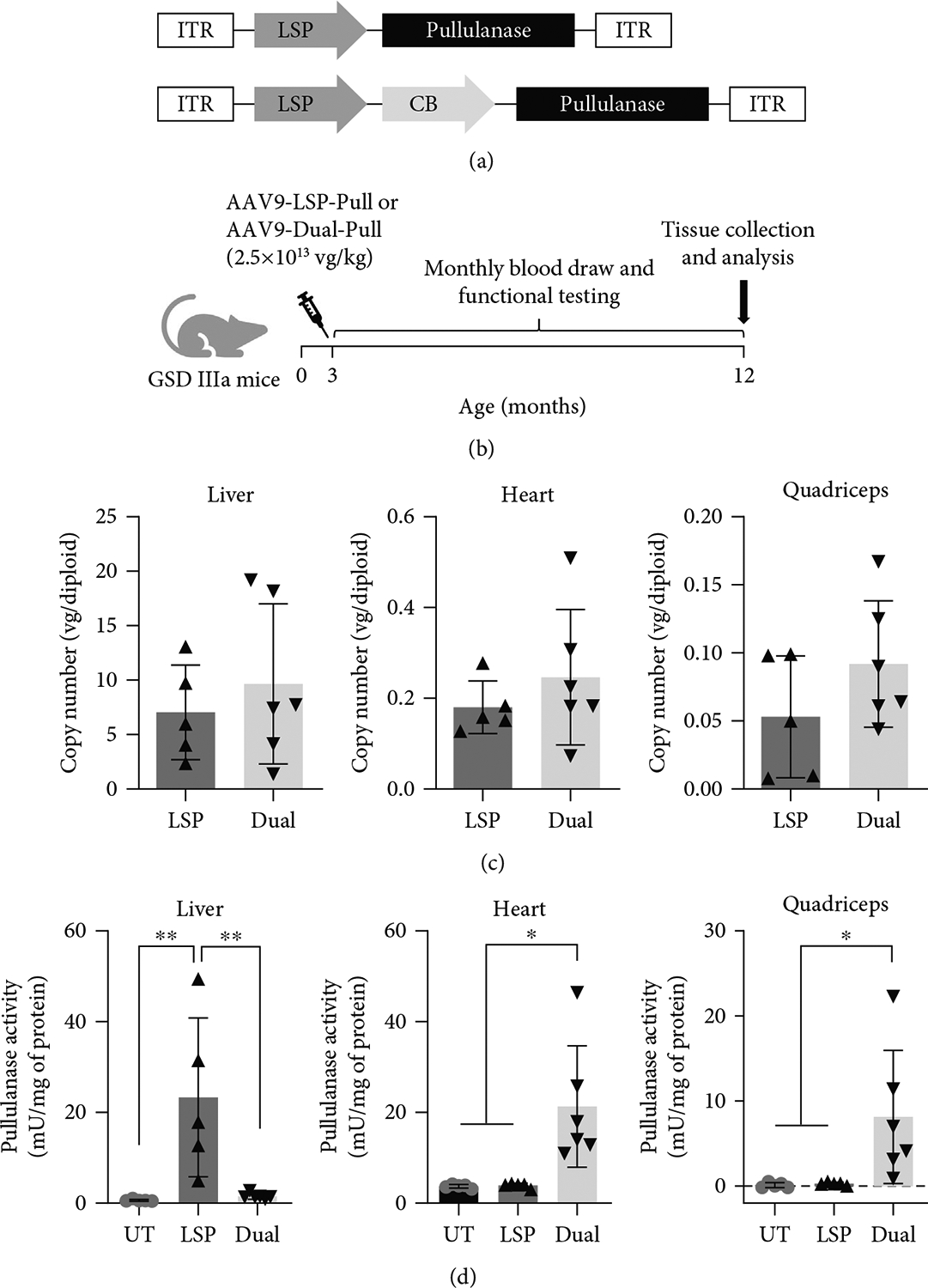

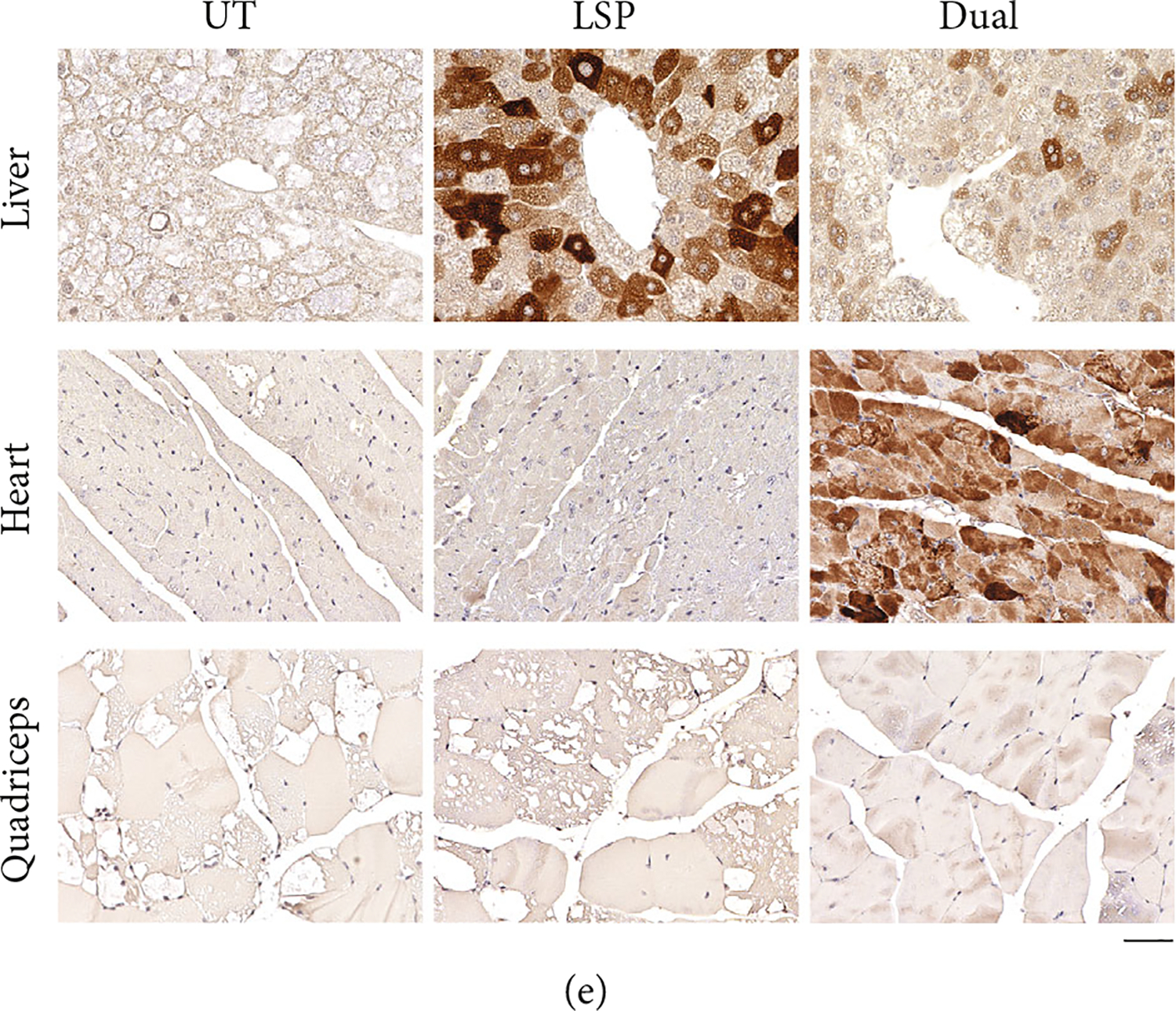
AAV9-Dual-Pull treatment allowed sustained pullulanase expression in major affected tissues of GSD IIIa mice. (a) The diagram shows the constructs of AAV vector containing the 2.2 kb codon-optimized pullulanase cDNA linked to a HA-tag at the 3′ end under the control of the liver-specific promoter (LSP) and the LSP-CB dual promoter. ITR: inverted terminal repeats; CB: CMV enhancer/chicken *β*-actin. (b) Three-month-old GSD IIIa mice were injected with AAV9-LSP-Pull or AAV9-Dual-Pull vector at a dose of 2.5 × 10^13^ vg/kg. Tissues were collected and analyzed 9 months after AAV injection. Plasma samples were collected and behavioral tests were performed monthly throughout the period of AAV treatment. (c) AAV genome copy numbers in the liver, heart, and quadriceps were determined by real-time PCR using specific primer pairs targeting pullulanase. There were no significant differences between AAV-LSP-Pull and Dual-Pull treatments. The graphs represent the mean ± SD. LSP, AAV9-LSP-Pull–treated group (*n* = 5); Dual, AAV9-Dual-Pull–treated group (*n* = 6). Each dot represents an individual mouse. Unpaired Student’s *t*-test, *p* > 0.05. (d) Pullulanase activities were measured in the liver, heart, and quadriceps. The enzyme activity was significantly elevated in the AAV-LSP-Pull–treated liver and AAV-Dual-Pull–treated heart and quadriceps. The graphs represent the mean ± SD. *n* = 5 for UT and AAV-LSP-Pull–treated groups; *n* = 6 for AAV-Dual-Pull–treated group. Each dot represents an individual mouse. Ordinary one-way ANOVA, *p < 0.05 and **p < 0.01. (e) Immunohistochemistry was performed to detect the expression of pullulanase in tissues. Pullulanase-expressed cells (brown) were detected in both the AAV-LSP-Pull–treated and AAV-Dual-Pull–treated liver (LSP > > Dual). Pullulanase was expressed highly in the AAV-Dual-Pull–treated heart and weakly in the AAV-Dual-Pull–treated quadriceps. No pullulanase-positive cells were observed in the AAV-LSP-Pull–treated heart and quadriceps. The images represent at least three mice in each group. Scale bar, 50 *μ*m.

Three-month-old GSD IIIa mice were intravenously injected with AAV9-LSP-Pull or AAV9-Dual-Pull at the same dose of 2.5 × 10^13^ vg/kg (Figure 1a,b). To determine the AAV vector biodistribution, we first performed quantitative PCR using primer pairs targeting pullulanase. Nine months after AAV injection, both AAV-LSP-Pull–treated and AAV-Dual-Pull–treated mice showed significant amounts of AAV genomes in the liver (LSP: 7.04 ± 4.35 vg/diploid genome; Dual: 9.66 ± 7.36), heart (LSP: 0.18 ± 0.06; Dual: 0.32 ± 0.23), and quadriceps (LSP: 0.05 ± 0.04; Dual: 0.09 ± 0.04) (Figure 1c).

We next measured the pullulanase expression in those tissues. In comparison with the UT mice, AAV-LSP-Pull–treated mice displayed significantly increased pullulanase activity in the liver but not in the heart or quadriceps; AAV-Dual-Pull–treated mice showed significantly increased pullulanase activity in the heart and quadriceps but not in the liver (Figure 1d). Since AAV9 can cross the brain–blood barrier (BBB) at low efficiency, the pullulanase activity in the brain was also examined. The result showed that neither AAV-LSP-Pull nor AAV-Dual-Pull treatment significantly increased the pullulanase activity in the brain (Figure S1A).

IHC staining of tissue sections with an anti-HA-tag antibody showed that the AAV-LSP-Pull treatment resulted in robust pullulanase expression in the liver, whereas no apparent expression of pullulanase was observed in the heart and quadriceps (Figure 1e); AAV-Dual-Pull–treated mice exhibited strong pullulanase expression in the heart and low-level expression in the quadriceps (Figure 1e). Interestingly, we observed a significant number of pullulanase-positive hepatocytes in the AAV-Dual-Pull–treated liver by IHC staining (Figure 1e), despite no significant increase in enzyme activity was detected in the liver homogenates (Figure 1d).

### 3.2. AAV-Dual-Pull Treatment Led to Long-Term Reduction of Glycogen Accumulation in Major Affected Tissues in GSD IIIa Mice.

**Figure 2:**
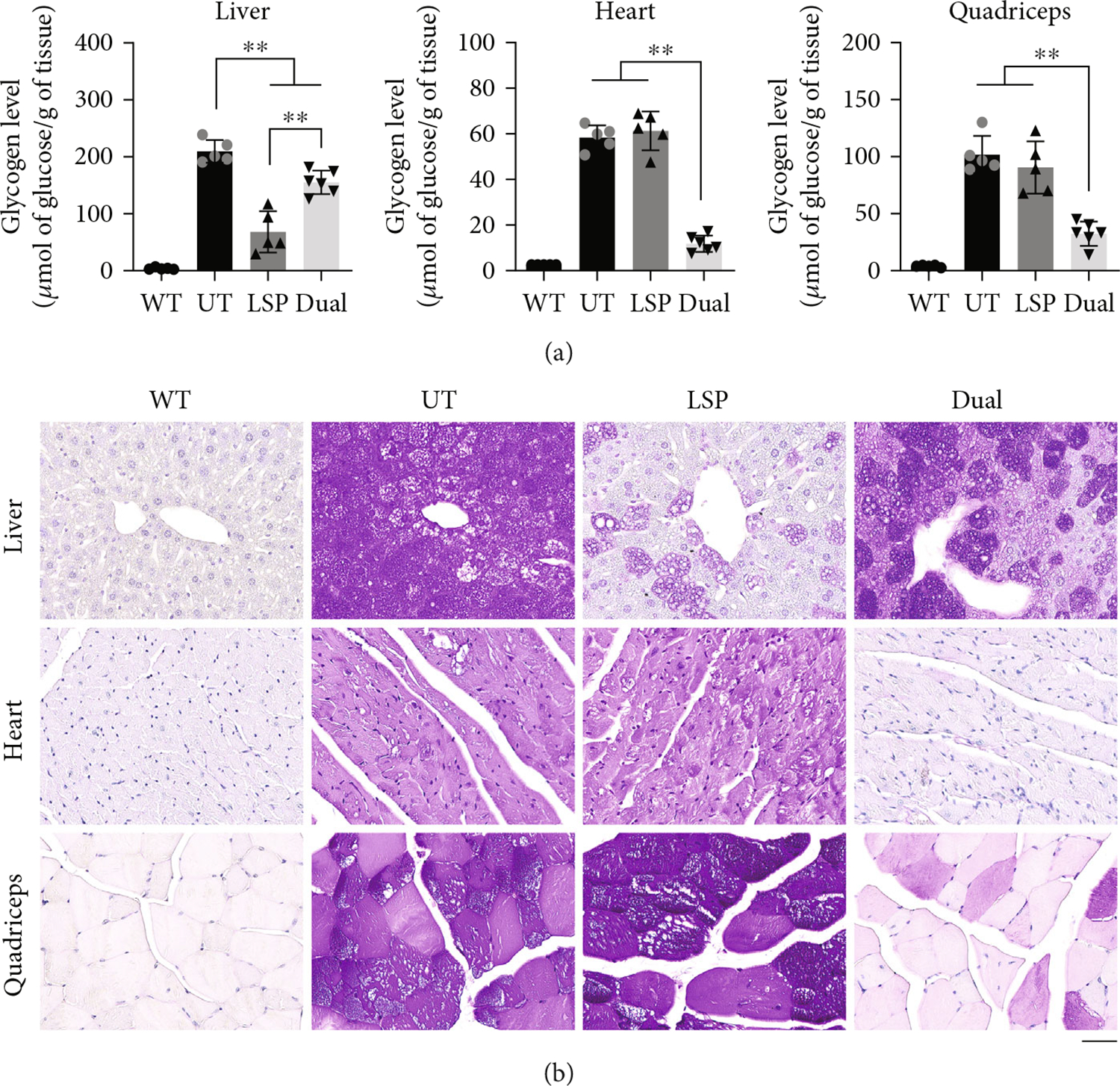
AAV-Dual-Pull treatment significantly reduced glycogen accumulation in the liver, heart, and skeletal muscle of GSD IIIa mice. (a) Glycogen contents were measured in the liver, heart, and quadriceps 9 months after AAV injection. Age-matched wild-type (WT) mice were included as normal controls. Glycogen level was significantly decreased in both AAV-LSP-Pull–treated and AAV-Dual-Pull–treated livers. Only the AAV-Dual-Pull treatment also significantly reduced glycogen levels in the heart and quadriceps. The graphs represent the mean ± SD. *n* = 5 for WT, UT, and AAV-LSP-Pull–treated groups; *n* = 6 for the AAV-Dual-Pull–treated group. Each dot represents an individual mouse. Ordinary one-way ANOVA (excluding WT data), **p < 0.01. (b) Periodic acid–Schiff (PAS) staining of tissue sections was performed to confirm the glycogen content results in (a). The UT mice showed heavily stained glycogen (purple) in the liver, heart, and quadriceps. The AAV-LSP-Pull–treated livers showed light staining of glycogen, and the glycogen staining was moderately decreased in the AAV-Dual-Pull–treated liver compared with the UT liver. Only the AAV-Dual-Pull treatment also remarkably cleared glycogen accumulation in the heart and quadriceps. No glycogen accumulation was observed in any tissues of WT mice. The images represent at least three mice in each group. Scale bar, 50 *μ*m.

We next assessed the glycogen contents in the liver, heart, quadriceps, and the brain at 9 months following AAV injection. In comparison with UT, both AAV-LSP-Pull and AAV-Dual-Pull treatments significantly decreased glycogen content in the liver (−67% by LSP and −33% by Dual), but only AAV-Dual-Pull treatment also significantly reduced glycogen levels in the heart (−80%) and quadriceps (−66%). In the brain, glycogen level did not change significantly by AAV-LSP-Pull or AAV-Dual-Pull treatment (Figure S1B). As expected, no glycogen accumulation was detected in any of the tissues of WT control mice (Figure 2a).

**Figure 3:**
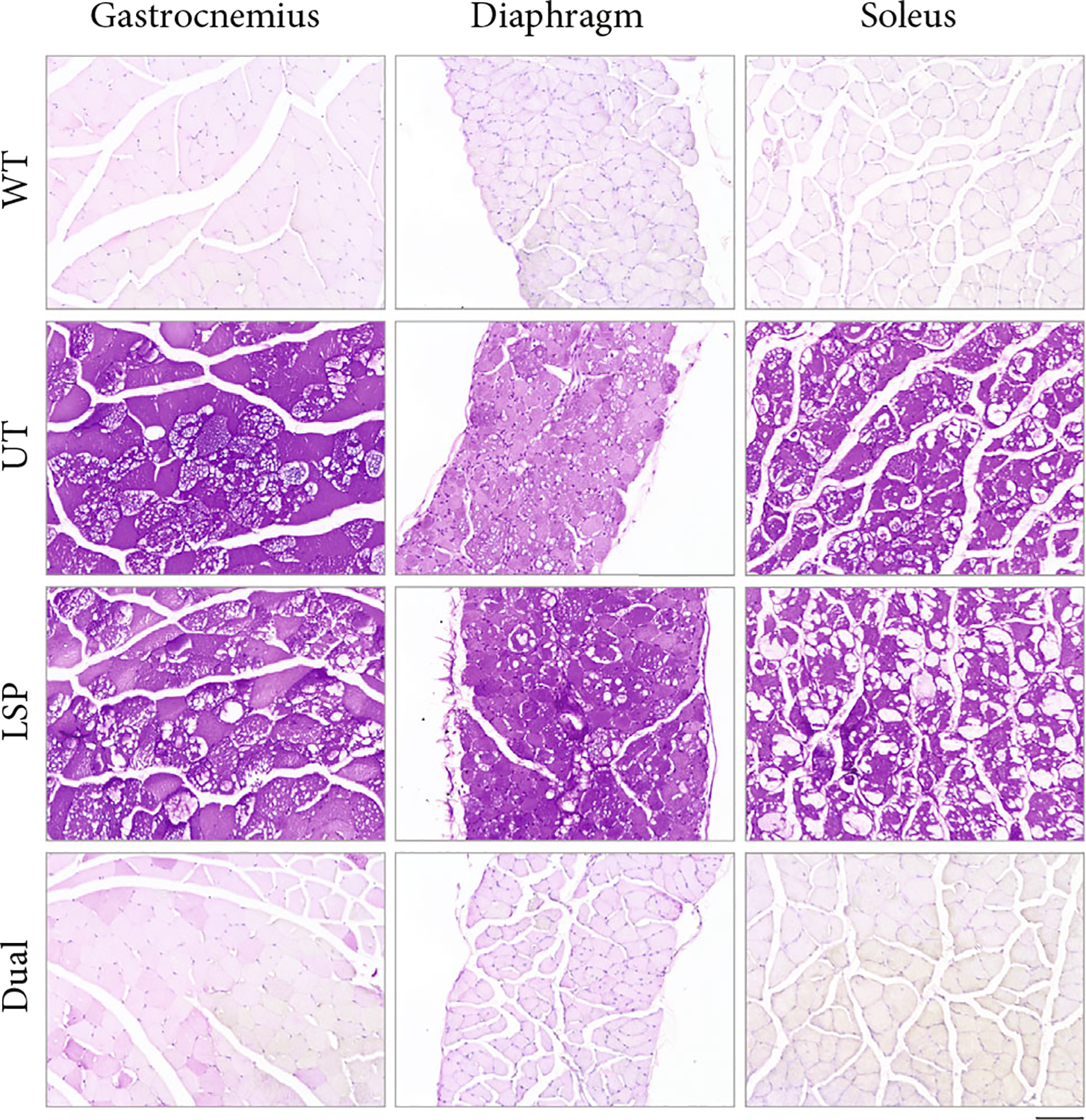
AAV-Dual-Pull treatment significantly cleared glycogen accumulation in other skeletal muscles. Glycogen clearance was assessed by PAS staining of gastrocnemius, diaphragm, and soleus sections from UT, AAV-LSP-Pull–treated, and AAV-Dual-Pull–treated GSD IIIa mice at 9 months following AAV administration. Age-matched WT mice were included as normal controls. The images represent at least three mice in each group. Scale bar, 100 *μ*m.

Periodic acid–Schiff (PAS) staining of glycogen in these tissues was conducted to confirm the glycogen content results. As shown in Figure 2b, intensive glycogen-positive cells (purple staining) were detected in all tested tissues of UT mice, whereas no glycogen staining was observed in any WT tissues. AAV-LSP-Pull treatment greatly decreased glycogen staining in the liver but had no effect on the heart or quadriceps; AAV-Dual-Pull treatment reduced glycogen staining moderately in the liver, remarkably in the quadriceps, and almost completely in the heart. Furthermore, the AAV-Dual-Pull treatment also reduced glycogen accumulation in other skeletal muscles including gastrocnemius, diaphragm, and soleus (Figure 3). These results demonstrated that long-term treatment with AAV-Dual-Pull successfully decreased glycogen accumulation in all major affected tissues of GSD IIIa mice.

### 3.3. Both AAV-LSP-Pull and AAV-Dual-Pull Treatments Normalized Liver Size and Prevented Hepatic Fibrosis in GSD IIIa Mice.

**Figure 4:**
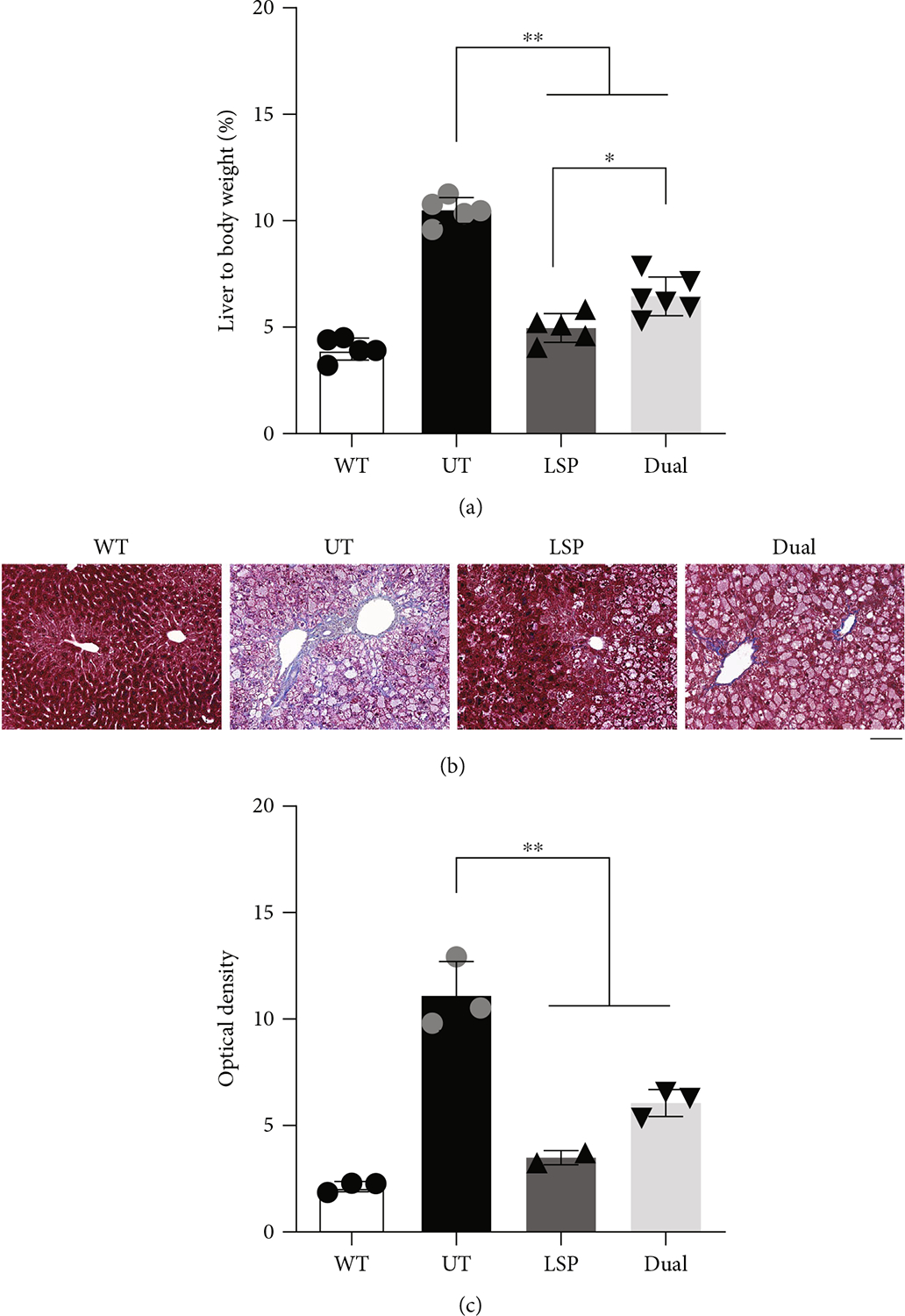
AAV-Dual-Pull treatment corrected liver abnormalities. (a) The liver-to-body weight ratio was measured to determine the liver size 9 months after AAV injection. The ratios were significantly decreased in both AAV-LSP-Pull–treated and AAV-Dual-Pull–treated mice in comparison with UT. The graphs represent the mean ± SD. *n* = 5 for WT, UT, and AAV-LSP-Pull–treated groups; *n* = 6 for AAV-Dual-Pull–treated group. Each dot represents an individual mouse. Ordinary one-way ANOVA (excluding WT data), *p < 0.05 and **p < 0.01. (b) Trichrome staining of liver sections was performed to detect liver fibrosis (blue). Both AAV-LSP-Pull and AAV-Dual-Pull treatments prevented liver fibrosis. At least three mice in each group were examined, and representative images are shown. Scale bar, 100 *μ*m. (c) Quantitative analysis of liver fibrosis by measuring the optical density of blue staining in each image. Three different areas of each liver section were examined. The graphs represent the mean ± SD. *n* = 3 for WT, UT, and AAV-Dual-Pull–treated groups; *n* = 2 for AAV-LSP-Pull–treated group. Each dot represents the mean of three independent areas. Ordinary one-way ANOVA (excluding WT data), *p < 0.05 and **p < 0.01.

Hepatomegaly and hepatic fibrosis are commonly seen in patients with GSD III [3]. Therefore, we measured the liver size by calculating the ratio of liver to body weight and evaluated the hepatic fibrosis by trichrome staining of liver sections of GSD IIIa mice. At 12 months of age (9 months after AAV injection), the liver size of UT GSD IIIa mice was 2.6-fold that of WT mice (Figure 4a). In contrast, both AAV-LSP-Pull and AAV-Dual-Pull treatments significantly reduced the liver size: the AAV-LSP-Pull–treated liver showed a similar size to the WT liver, while the AAV-Dual-Pull–treated liver was slightly larger than the AAV-LSP-Pull–treated liver (Figure 4a). Trichrome staining of liver sections showed massive pericellular and periportal fibrosis (blue staining) in the UT liver, negligible in the AAV-treated livers, and invisible in the WT liver (Figure 4b and Figure S2). Quantification of the blue-stained fibrotic tissues in the liver sections by ImageJ software showed that the UT liver had high level of liver fibrosis, while AAV-LSP-Pull and AAV-Dual-Pull–treated livers displayed significantly lowered fibrosis (Figure 4c).

### 3.4. AAV-Dual-Pull Treatment Decreased Liver and Muscle Enzyme Activities in Plasma, As Well As the Disease Biomarker Glc4 in the Urine of GSD IIIa Mice.

**Figure 5:**
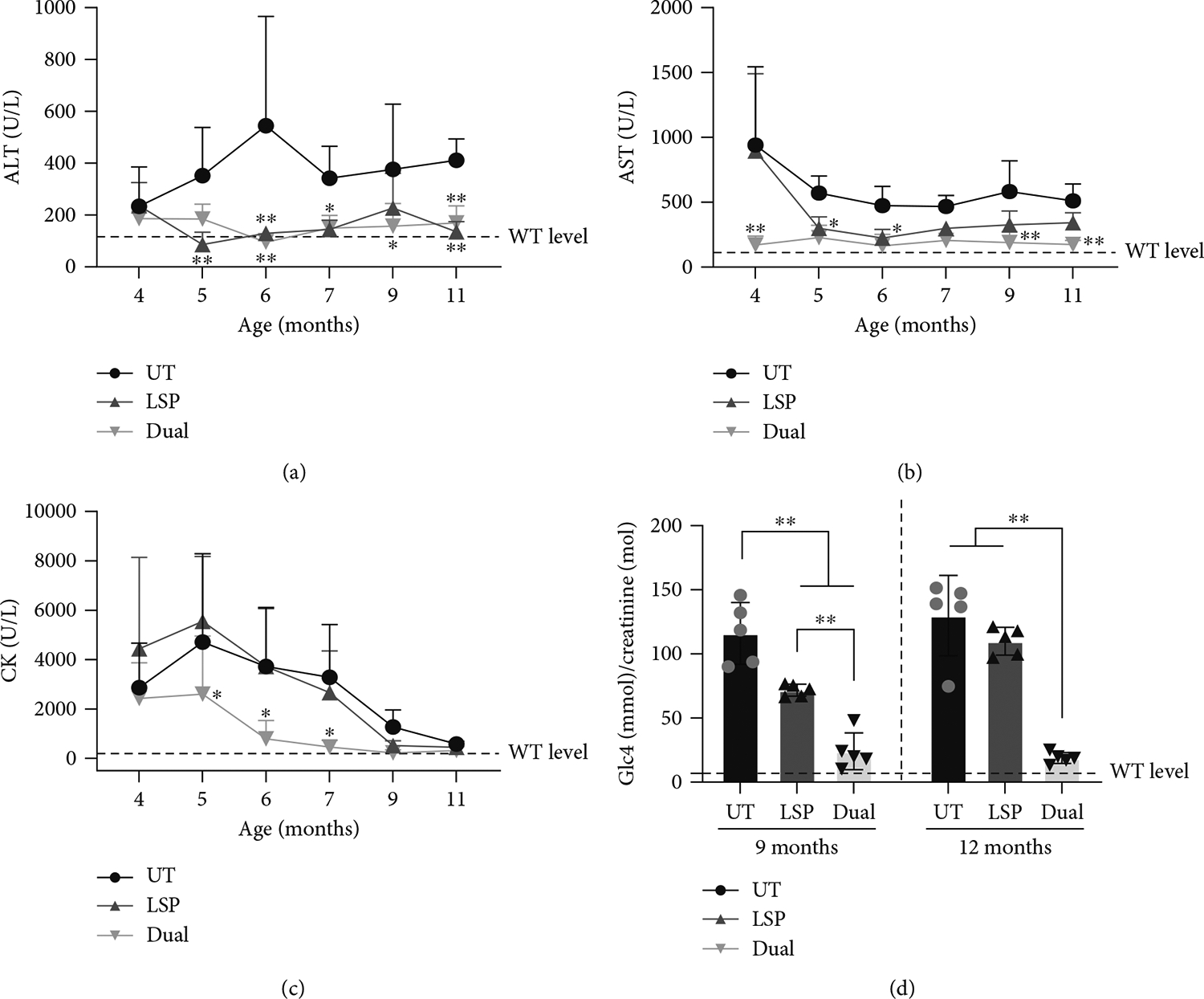
AAV-Dual-Pull treatment decreased liver and muscle enzyme activities in plasma and normalized disease biomarker levels in urine. (a–c) Plasma ALT, AST, and CK activities were measured monthly during AAV treatment from 4 to 11 months of age (corresponding to 1–8 months following AAV injection) to evaluate the liver and muscle damage. Both AAV-LSP-Pull–treated and AAV-Dual-Pull–treated mice showed significantly decreased ALT levels, but only AAV-Dual-Pull–treated mice also showed significant reduction in AST and CK levels compared with UT. The graphs represent the mean ± SD. *n* = 6 (CK) or 7 (ALT and AST) for UT, *n* = 5 for WT and AAV-LSP-Pull–treated groups, and *n* = 6 for the AAV-Dual-Pull–treated group. The dotted line represents the WT levels. Two-way ANOVA (excluding WT data), *p < 0.05 and **p < 0.01. Black asterisks indicate the significance of the AAV-LSP-Pull–treated group versus UT; gray asterisks indicate the significance of the AAV-Dual-Pull–treated group versus UT. (d) The concentrations of urinary Glc4 were assessed by mass spectrometry [27]. Urinary Glc4 level in the AAV-LSP-Pull–treated group was significantly reduced at 9 months of age but returned to UT level at 12 months of age. Urinary Glc4 level in the AAV-Dual-Pull–treated group was significantly lower than that in the UT or AAV-LSP-Pull–treated mice at both 9 and 12 months of age. The urinary creatinine level was used for normalization. The graph represents the mean ± SD. *n* = 5 for all groups. Each dot represents an individual mouse. The dotted line represents the WT level. Two-way ANOVA (excluding WT data), **p < 0.01.

Because ALT, AST, and CK levels in blood were highly elevated in UT GSD IIIa mice [22], plasma ALT, AST, and CK levels were monitored monthly following AAV injection. In comparison with UT, both AAV-LSP-Pull and AAV-Dual-Pull treatments significantly reduced plasma ALT activity to the WT level (Figure 5a), but only AAV-Dual-Pull treatment also significantly decreased AST (Figure 5b) and CK levels (Figure 5c) throughout the treatment period. We next measured the urinary Glc4 level, which has been suggested as a biomarker for GSD III [28, 29]. As shown in Figure 5d, there was no significant increase in urinary Glc4 level from age 9 to 12 months (corresponding to 6–9 months following AAV injection) in the UT mice. Urinary Glc4 level in the AAV-LSP-Pull–treated mice was significantly lower than that in the UT mice at 9 months of age but returned to the UT level at 12 months of age. AAV-Dual-Pull treatment significantly reduced urinary Glc4 to near the WT level at both time points.

### 3.5. AAV-Dual-Pull Treatment Improved Muscle Function in GSD IIIa Mice.

**Figure 6:**
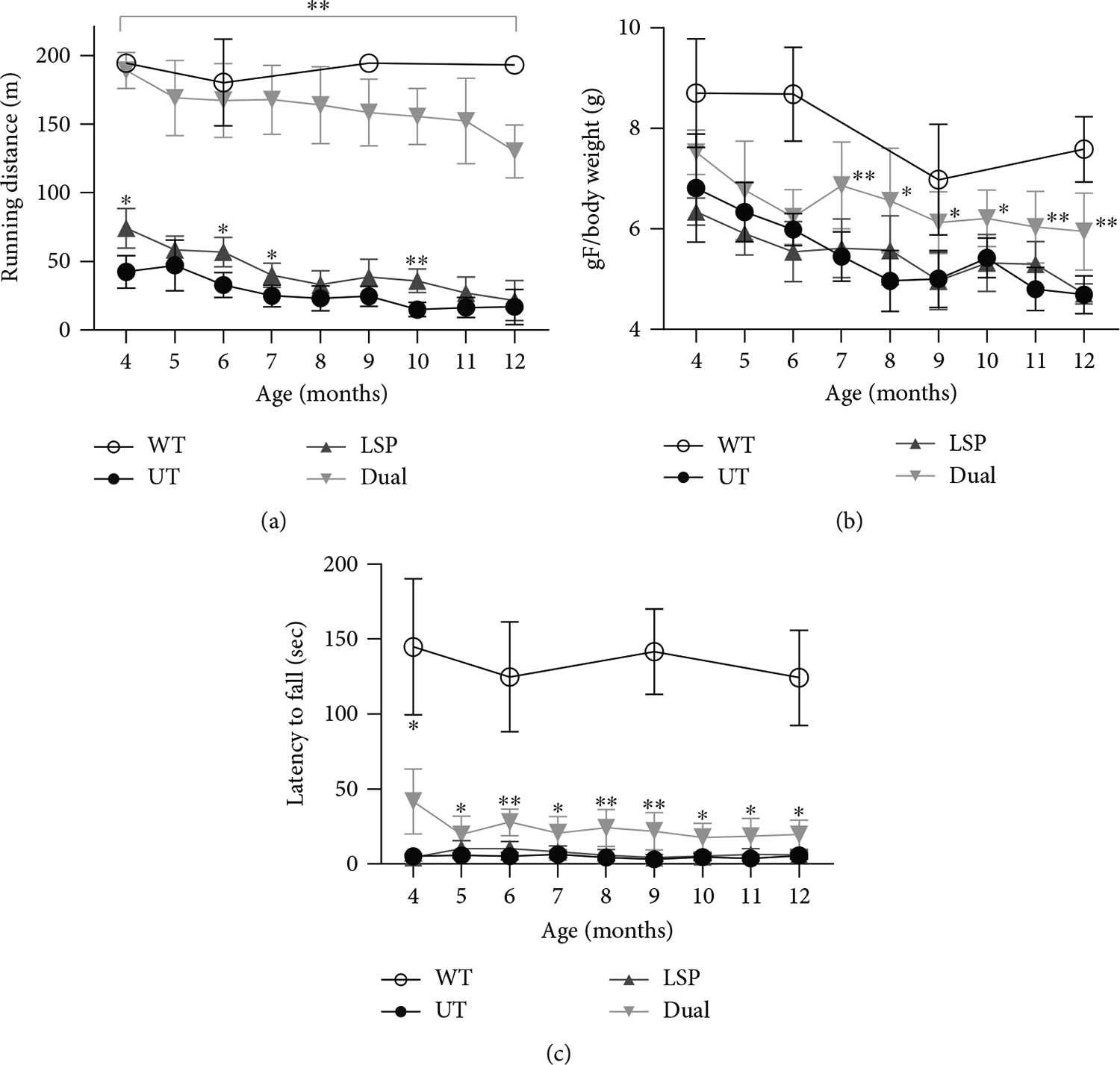
AAV-Dual-Pull treatment improved muscle function. Treadmill (a), grip strength (b), and wire hang (c) tests were performed monthly to evaluate muscle function throughout the AAV treatment period. Only the AAV-Dual-Pull treatment significantly improved all the performances compared with UT. The graphs represent the mean ± SD. *n* = 5 for WT and AAV-LSP-Pull–treated groups; *n* = 6 for UT and AAV-Dual-Pull–treated groups. Two-way ANOVA (excluding WT data), *p < 0.05 and **p < 0.01. Black asterisks indicate the significance of the AAV-LSP-Pull–treated group versus UT; gray asterisks indicate the significance of the AAV-Dual-Pull–treated group versus UT.

To evaluate the therapeutic effect of AAV-Dual-Pull on muscle function, we performed monthly tread-mill, grip strength, and wire hang tests over the course of AAV treatment. AAV-LSP-Pull–treated mice displayed impaired treadmill performance as the UT mice, although there were slight increases at some time points; in contrast, AAV-Dual-Pull–treated mice showed remarkably increased running distance at all tested time points, although the running distance slightly decreased over time (Figure 6a). In the grip strength test, no significant difference was observed between UT and AAV-LSP-Pull–treated mice throughout the treatment period; in contrast, AAV-Dual-Pull–treated mice showed significant improvement in grip strength from 7 to 12 months of age (Figure 6b). In the wire hang test, AAV-LSP-Pull treatment did not improve the hanging time, while AAV-Dual-Pull treatment resulted in a significant increase in hanging time (Figure 6c). These results demonstrated that AAV-Dual-Pull treatment achieved significant long-term improvement of muscle function in GSD IIIa mice.

## 4. Discussion

Gene therapy with recombinant AAV9 provides an ideal treatment for the correction of both liver and muscle diseases in GSD III. We recently reported a 10-week gene therapy study by intravenous injection of a single dose of an AAV9 vector expressing pullulanase driven by the LSP promoter (AAV9-LSP-Pull) or the LSP-CB dual promoter (AAV9-Dual-Pull) in adult GSD IIIa mice [22]. We demonstrated that the LSP promoter almost completely suppressed pullulanase-specific CTL responses and allowed persistent pullulanase expression and glycogen clearance in only the liver; in contrast, the Dual promoter effectively decreased pullulanase-specific CTL responses, to a lesser extent than the LSP promoter, and enabled pullulanase expression and glycogen reduction in all major affected tissues including the liver, heart, and skeletal muscle [22]. Here, we extended the study to examine the long-term efficacy of this gene therapy approach in adult GSD IIIa mice. Considering the significant loss of the vector genomes from 2 to 10 weeks in the AAV-transduced tissues in the previous study [22], we increased the vector administration dose to 2.5 × 10^13^ vg/kg in this study.

Nine months after AAV injection, the AAV-LSP-Pull–treated and AAV-Dual-Pull–treated mice had comparable amounts of AAV genomes in the liver, heart, and skeletal muscle (quadriceps) (Figure 1c). However, the AAV-LSP-Pull–treated liver showed significantly higher pullulanase expression (Figure 1d,e) and greater glycogen reduction (Figure 2a,b) than the AAV-Dual-Pull–treated liver, suggesting that AAV-LSP-Pull is a better therapeutic vector for gene therapy in patients with GSD IIIb that affects only the liver. It is worth noting that even though no obviously increased pullulanase activity was observed in the liver homogenates of the AAV-Dual-Pull treated mice (Figure 1d), pullulanase expression was detected in significant numbers of hepatocytes by IHC staining of the liver sections (Figure 1e). This discrepancy was likely caused by the colored liver homogenates (blood contamination) that interfered with the sensitivity of the pullulanase activity assay used in this study, based on our experience.

The sustained expression of pullulanase in the liver, heart, and skeletal muscle by the AAV-Dual-Pull treatment significantly reduced glycogen accumulation in these tissues (Figures 2 and 3), rescued liver abnormalities (Figure 4), decreased plasma enzyme activities (Figures 5a, 5b, and 5c), normalized disease biomarker urinary Glc4 (Figure 5d), and improved muscle function (Figure 6), suggesting that AAV-Dual-Pull is an effective gene therapy approach for patients with GSD IIIa that affects both liver and muscle. As expected, this treatment did not correct glycogen storage in the brain (Figure S1B) and spinal cord and smooth muscle (data not shown), which was consistent with our previously published results [22].

Gradual decrease of elevated plasma ALT, AST, and CK activities due to disease progression had been shown in GSD IIIa patients and dogs [30, 31]; here, we demonstrated that UT GSD IIIa mice displayed a similar trend in plasma AST and CK activities (Figure 5b,c). For example, the plasma CK activity in the UT GSD IIIa mice peaked at 5 months of age and then gradually decreased to the WT level at 11 months of age (Figure 5c). Most importantly, gene therapy with AAV-Dual-Pull significantly reduced the plasma ALT, AST, and CK levels throughout the course of treatment (Figures 5a, 5b, and 5c).

## 5. Conclusion

In conclusion, our results demonstrate that gene therapy with a single dose of the AAV-Dual-Pull vector allows sustained expression of pullulanase and long-term clearance of glycogen accumulation in liver, heart, and skeletal muscle of GSD IIIa mice, while AAV-LSP-Pull treatment shows a better treatment efficacy in liver but has no effect on cardiac and skeletal muscles. Although this study shows promising long-term therapeutic outcomes in GSD IIIa mice, further studies are needed to test this gene therapy approach in large animal models, such as GSD IIIa dogs, which are currently underway. As glycogen storage diseases like GSD IIIa affect multiple organs from early development, including prenatal stages, it will be interesting to see whether earlier gene therapy intervention, such as prenatal or immediate postnatal treatment, would be more effective in correcting glycogen storage and alleviating disease progression.

## Supplementary Material

Liao et al., Suppl.

Additional supporting information can be found online in the Supporting Information section. (Supporting Information) Figure S1 shows that both the AAV-LSP-Pull and AAV-Dual-Pull treatments had no effect on the brain. (A) Pullulanase activity was not detectable in the brain of AAV-LSP-Pull–treated or AAV-Dual-Pull–treated mice. (B) Glycogen content remained unchanged in the brain after AAV-LSP-Pull or AAV-Dual-Pull treatment. The graphs represent the mean ± SD. *n* = 5 for UT and AAV-LSP-Pull–treated groups, *n* = 6 for AAV-Dual-Pull–treated group. Each dot represents an individual mouse. Ordinary one-way ANOVA (excluding WT data), *p* > 0.05. Figure S2 shows that the AAV-Dual-Pull treatment reversed liver fibrosis. Trichrome staining was used for the detection of liver fibrosis. Immense size of fibrotic tissues (blue) was observed in the untreated liver, but AAV-LSP-Pull and AAV-Dual-Pull–treated liver showed reduced fibrosis. At least three mice in each group were examined and representative images are shown. Scale bar, 1000 *μ*m.

## Data Availability

The data that support the findings of this study are available from the corresponding author upon reasonable request.
