# Supplementary material for: Long-Term Correction of Murine Glycogen Storage Disease Type III by AAV-Mediated Gene Therapy Using an Immunotolerizing Dual Promoter to Express Bacterial Pullulanase
Source: Adv Cell Gene Ther. Author manuscript; Available in PMC 2025 Oct 7. (PMC12499360; doi:10.1155/acg2/4639392)
Supplement: Liao et al., Suppl. [file NIHMS2114611-supplement-Liao_et_al___Suppl_.pdf]

Supplementary Materials

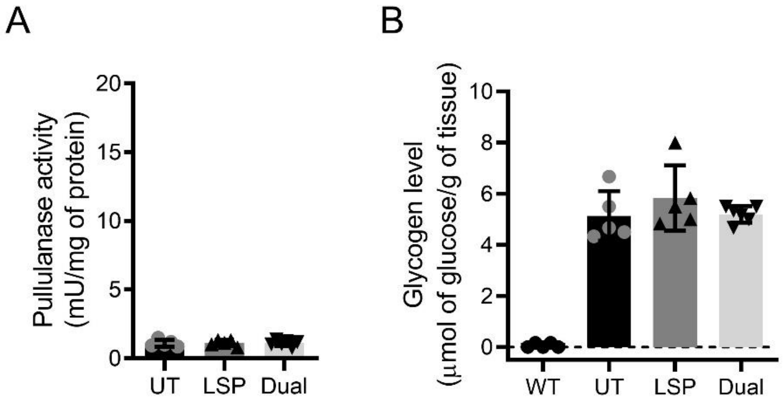

Figure S1. Both AAV-LSP-Pull and AAV-Dual-Pull treatments had no effect on the brain. (A) Pullulanase activity was not detectable in the brain of AAV-LSP-Pull-treated or AAV-Dual-Pull-treated mice. (B) Glycogen content remained unchanged in the brain after AAV-LSP-Pull or AAV-Dual-Pull treatment. The graphs represent the mean ± SD. n=5 for UT and AAV-LSP-Pull-treated groups, n=6 for AAV-Dual-Pull-treated group. Each dot represents an individual mouse. Ordinary one-way ANOVA (excluding WT data), p>0.05.

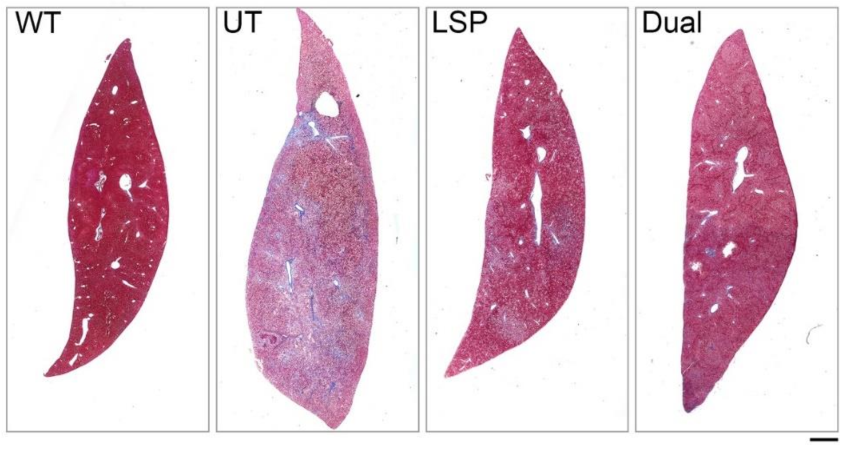

Figure S2. AAV-Dual-Pull treatment reversed liver fibrosis. Trichrome staining was used for the detection of liver fibrosis. Immense size of fibrotic tissues (blue) were observed in the untreated liver, but AAV-LSP-Pull and AAV-Dual-Pull-treated liver showed reduced fibrosis. At least three mice in each group were examined and representative images are shown. Scale bar, 1000 μm.
